# Coastal Bacterioplankton Metabolism Is Stimulated Stronger by Anthropogenic Aerosols than Saharan Dust

**DOI:** 10.3389/fmicb.2017.02215

**Published:** 2017-11-15

**Authors:** Isabel Marín, Sdena Nunes, Elvia D. Sánchez-Pérez, Estibalitz Txurruka, Carolina Antequera, Maria M. Sala, Cèlia Marrasé, Francesc Peters

**Affiliations:** Institut de Ciències del Mar (CSIC), Barcelona, Spain

**Keywords:** Saharan dust, anthropogenic aerosols, bacterial production, enzymatic activity, coastal areas, Mediterranean Sea, marine bacteria

## Abstract

In oligotrophic regions, such as the Mediterranean Sea, atmospheric deposition has the potential to stimulate heterotrophic prokaryote growth and production in surface waters, especially during the summer stratification period. Previous studies focused on the role of leaching nutrients from mineral particles of Saharan (S) origin, and were restricted to single locations at given times of the year. In this study, we evaluate the effect of atmospheric particles from diverse sources and with a markedly different chemical composition [S dust and anthropogenic (A) aerosols] on marine planktonic communities from three locations of the northwestern Mediterranean with contrasted anthropogenic footprint. Experiments were also carried out at different times of the year, considering diverse initial conditions. We followed the dynamics of the heterotrophic community and a range of biogeochemical and physiological parameters in six experiments. While the effect of aerosols on bacterial abundance was overall low, bacterial heterotrophic production was up to 3.3 and 2.1 times higher in the samples amended with A and S aerosols, respectively, than in the controls. Extracellular enzymatic activities [leu-aminopeptidase (AMA) and β-glucosidase (β-Gl)] were also enhanced with aerosols, especially from A origin. AMA and β-Gl increased up to 7.1 in the samples amended with A aerosols, and up to 1.7 and 2.1 times, respectively, with S dust. The larger stimulation observed with A aerosols might be attributed to their higher content in nitrate. However, the response was variable depending the initial status of the seawater. In addition, we found that both A and S aerosols stimulated bacterial abundance and metabolism significantly more in the absence of competitors and predators.

## Introduction

Marine heterotrophic prokaryotes (hereafter referred to as “bacteria”) play fundamental roles in the oceanic part of the carbon cycle (Jiao et al., 2010). In oligotrophic regions, such as the Mediterranean Sea, most of the organic carbon (OC) is recycled in the surface layer and rapidly re-exchanged with the atmosphere (Moutin and Raimbault, 2002). Once the most labile fraction of OC is exhausted, bacteria have to synthetize extracellular enzymes in order to be able to hydrolyze polymeric substrates and then take up the monomers produced (Chróst, 1990). These processes are dependent on the availability of inorganic nutrients to bacteria inhabiting the surface waters. During the summer stratification period, large amounts of dissolved OC have been observed in Mediterranean surface waters, which has been attributed to a “malfunctioning” of the microbial loop, brought forth by nutrient limitation (mainly phosphorous, P, or P along with nitrogen, N), or competition between heterotrophic bacteria and phytoplankton (Thingstad et al., 1997; Pinhassi et al., 2006; Romera-Castillo et al., 2013). Instead, during periods of winter mixing – when inorganic nutrients are more abundant – bacterioplankton growth and production has been found to be limited in OC (Thingstad et al., 1997; Pinhassi et al., 2006).

The Mediterranean Sea is a quasi-enclosed basin, where external sources of nutrients have the potential to stimulate plankton growth and new production in surface waters. Changes in the relative importance of these external sources influence global oceanic C sequestration and affect CO_2_ air–sea exchange (Bréviere et al., 2015). Among these sources, atmospheric deposition is considered an increasingly important source of new nutrients to the coastal ocean (Duce et al., 1991; Paerl, 1995; Durrieu de Madron et al., 2011). In particular, Saharan (S) dust deposition has been shown to play a fertilizing effect on marine planktonic microorganisms (both autotrophic and heterotrophic) in oligotrophic areas, such as the Mediterranean, the Sargasso Sea, or the north Atlantic, especially during stratification conditions of the water column (Herut et al., 2005; Duarte et al., 2006; Marañón et al., 2010; Mackey et al., 2012; Guieu et al., 2014; Gallisai et al., 2016, 2014). The chemical composition of S dust over the Mediterranean atmosphere has been broadly characterized, being silicates and aluminosilicates the main components (Guieu et al., 2002; Moreno et al., 2006; Escudero et al., 2011; Nava et al., 2012). In comparison, anthropogenic (A) particles in the Mediterranean atmosphere are richer in N, sulfate, and organic compounds, as well as trace metals (Guerzoni et al., 1999; Pateraki et al., 2012; Moreno et al., 2013). In the northern hemisphere, the main sources of atmospheric N are anthropogenic (Guerzoni et al., 1999). Both mineral and A aerosols are also a source of P to surface waters (Migon et al., 2001; Krom et al., 2004). However, most of the P in S dust is present as apatite, being poorly soluble in seawater (Atlas and Pytkowicz, 1977). In contrast, P and trace metals delivered from A sources are more acidic and thus more soluble in seawater, what makes them more available for planktonic communities (Migon et al., 2001; Nenes et al., 2011; Jickells and Moore, 2015; Stockdale et al., 2016).

The role of S dust on marine microbial communities has been assessed through different experiments in the oligotrophic Mediterranean Sea (e.g., Herut et al., 2005; Guieu et al., 2010, 2014; Lekunberri et al., 2010). In spite of the potentially higher fertilizing effect of A aerosols with respect to mineral aerosols in the oligotrophic ocean, their impact on marine heterotrophic bacteria remains poorly studied. Malits et al. (2015) assessed the effect of black carbon on marine bacteria in the North Sea and found an increase in bacterial biomass and production. In the Mediterranean Sea, a couple of studies have evaluated the effect of A atmospheric particles on bacterial abundance (Bonnet et al., 2005; Ternon et al., 2011), showing almost no response. Bacterial production shows a higher sensitivity to external changes compared to bacterial abundance (Gasol and Duarte, 2000). However, to our knowledge, the effect of A aerosols on marine bacterial production in the Mediterranean has only been assessed in one location during the stratification period (Herut et al., 2016). In the same study, Krom et al. (2016) tested the effect of both dust and A aerosols on the activity of alkaline phosphatase (APA), an extracellular enzyme mainly produced by algae to split phosphorous from organic matter, whereas no study has focused on the effect of aerosols on other extracellular enzymes that are mainly synthesized by bacteria, as aminopeptidases and glucosidases (Sala et al., 2010, and references therein).

Moreover, it is still under discussion whether bacteria take up nutrients leached directly from the aerosols or if it is the growth of phytoplankton that induces an indirect stimulation of the heterotrophic community. Previous aerosol-amendment experiments have focused their attention either on the effect of atmospheric particles on the bacterial community only, without competitors or predators (Pulido-Villena et al., 2008) or with phytoplankton and grazers altogether (Herut et al., 2005; Lekunberri et al., 2010; Pulido-Villena et al., 2014), being difficult to estimate the magnitude of the direct effect of atmospheric particles on bacteria. To properly assess the effect of both S dust and anthropogenically derived aerosols on bacteria, we performed a parallel experiment with and without phytoplankton and bacterial predators.

Most of the studies that assess the effect of aerosols on the marine planktonic community are reduced to single locations or performed at a given time of the year. In the Mediterranean Sea – and in most water bodies located in midlatitudes – the initial biogeochemical conditions in the seawater may differ during the year and between locations. Hence, we evaluated the effect of both types of atmospheric particles at two coastal locations of the NW Mediterranean with a different A footprint (Barcelona, urban location, and Blanes, less impacted by human activities) and in open ocean waters offshore the Balearic Islands. We also explored the effect of aerosols at different times of the year in Barcelona. We hypothesize that (i) a larger stimulation of bacterial growth and activity will occur with A aerosols compared to S dust, as the former are richer in inorganic and organic soluble compounds; (ii) a larger response of bacteria to aerosols is expected in the most oligotrophic location (offshore waters), as the initial nutrient concentration will be lower. As nutrient concentration will also be lower during the stratification period, we expect a larger response in late spring and summer compared to winter in Barcelona; and (iii) we expect a direct and higher effect of aerosols on bacterial growth and activity in the absence of competitors and predators.

## Materials and Methods

### Aerosol Collection

Total suspended particles (TSP) were sampled during dry atmospheric conditions (i.e., no rain) on quartz fiber filters (Munktell, Falun, Sweden). Particles were collected with a CAV-A/mb high volume sampler (MCV, Barcelona, Spain) operating at 30 m^3^ h^-1^ during 24 h. The device was located at the roof of the Institut de Ciències del Mar of Barcelona (41.39° N, 2.20° E; approximately 12 m AGL) and of the Centre d’Estudis Avançats of Blanes (41.68° N, 2.80° E; approximately 10 m AGL). Particulate matter trapped in the collected filters was gravimetrically determined, and the filters were subsequently cut in two equal pieces. One half of each filter was used for chemical composition analyses, and the other half was employed for inoculation into the experimental containers. “S” and “A” aerosols were classified based on advanced event warnings for the north-eastern (NE) Iberian Peninsula^1^, and the element ratios criteria defined in Marín et al. (2017). Atmospheric particles were recovered by submerging the filters in 250 ml of a 37‰ NaCl solution (EMSURE, Grade ACS, MERCK, Darmstadt, Germany) and after sonication in a Bandelin SONOREX Digital 10 P Ultrasonic bath (MERCK, Darmstadt, Germany) at 7 kHz. Once particles were released into the solution, filters were removed and we kept the solution in the freezer (-80°C) until being used in the microcosm experiments.

### Experimental Design and Analytical Procedures

#### Seawater Collection and Microcosm Set-up

**FIGURE 1:**
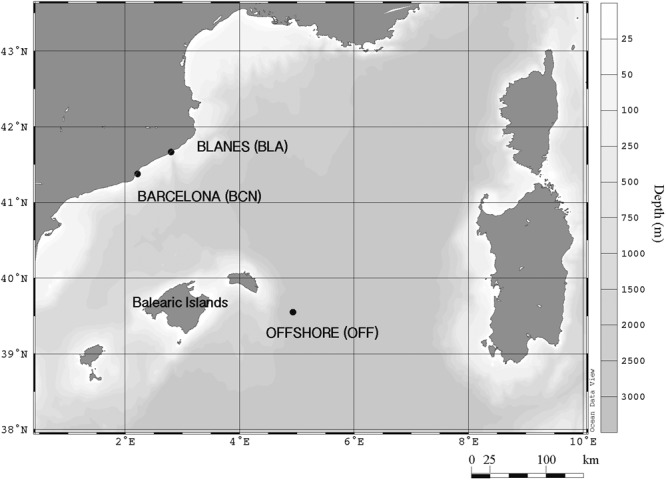
Geographical location of the two coastal sampling stations (BCN and BLA) and the OFF station, in the northwestern Mediterranean Sea.

Water was collected ca. 1 km from the coast of Barcelona (BCN) and Blanes (BLA), and offshore Balearic Islands (OFF), in the NW Mediterranean Sea (**Figure 1**). We carried out three experiments in BCN (late summer [SU] of 2013, and winter [WI] and spring [SP] of 2014), two in BLA (SP and SU of 2014), and one experiment offshore (late SU of 2014). In the experiments of BCN and BLA, surface water sieved through a 150-μm-nylon mesh was collected in acid-cleaned carboys and taken to the laboratory at the Institut de Ciències del Mar within 2 h. Seawater was distributed into 15-l acid-cleaned, methacrylate containers that were incubated in a temperature-controlled chamber at the *in situ* surface water temperature and subjected to the corresponding photoperiod at that time of the year. OFF instead was performed on board the R/V *García del Cid* and seawater was distributed into 10-l acid-cleaned, polypropylene carboys. Experiments lasted between 2 (OFF) and 6 days (BLA; SP) after the aerosol addition.

#### Aerosol Additions

Atmospheric particles of A or S origin were added at 0.8 mg l^-1^ into duplicate experimental containers (A and S, respectively). The aerosols were added as unique doses and containers were subsequently stirred with a sterile glass stick to homogenize particle distribution. Blank treatments were carried out (C), containing either no particles or seawater with blank sonicated filters as in A and S. These two types of control showed no significant differences between them (Marín et al., 2017). In the WI experiment instead, due to the higher frequency of pollution episodes of A origin from Europe (Viana et al., 2005; Pey et al., 2010), we decided to have two A aerosol treatments of different chemical composition. Aerosols used for each experiment were collected at the corresponding location (i.e., BCN or BLA), with the exception of OFF. Here, aerosols from BCN were used for the amendment, as there was no time to previously collect the aerosols offshore. With the aim to evaluate the effect of aerosols on bacteria considering no grazers (mainly heterotrophic nanoflagellates) or competitors (phytoplankton), in the SU experiment of BCN, we carried out the same set-up with seawater filtered by 0.8 μm to remove all cells but picoplankton, leaving basically heterotrophic prokaryotes behind (treatments CF, AF, and SF hereafter).

#### Biochemical Sampling and Analyses

Samples were taken from each microcosm by siphoning water through milli-Q rinsed and autoclaved glass tubes. Duplicate containers for each of the three treatments were simultaneously and independently sampled. Sampling for inorganic nutrients (nitrate, nitrite, ammonium, and phosphate), total organic carbon (TOC), total organic phosphorous (TOP), heterotrophic bacterial abundance (HBA), heterotrophic bacterial production (HBP), and extracellular enzymatic activity was performed daily. In addition, analyses of fluorescent dissolved organic matter (FDOM) were carried out before and after the aerosol additions, in order to assess the quality of the organic matter supplied by both types of atmospheric particles. All these variables were measured following standard procedures that can be found elsewhere. Briefly, 10 ml samples of inorganic nutrients were measured with an Auto Analyzer AA3 HR (SEAL Analytical, Norderstedt, Germany) following the methods described in Grasshoff et al. (1999). For TOC determination, 10 ml samples were collected in pre-combusted glass ampoules (450°C, 24 h), acidified to pH < 2 and stored in the dark until analyzed with a TOC-5000 analyzer (SHIMADZU, Kioto, Japan) following the high temperature catalytic oxidation (HTCO) technique described by Cauwet (1994). The system was calibrated daily with a solution of acetanilide. TOP was calculated by extracting inorganic phosphorous to total phosphorous. Total phosphorous was analyzed following the wet oxidation and colorimetric analysis described by Hansen and Koroleff (1999). Samples of 25 ml were mixed with 2 ml of an oxidant reagent in glass vials and autoclaved at 121°C for 30 min. Once the vials were cooled down to room temperature, 556 μl of ascorbic acid and the same amount of a combined reagent were added. The samples were kept in the dark for 15 min before analysis in a CaryWin UV Spectrophotometer (VARIAN, Palo Alto, CA, United States). Readings were done at 880 nm. FDOM was measured after temperature acclimation according to Nieto-Cid et al. (2006). Single measurements of seawater and emission–excitation (Em/Ex) matrices were performed with a Perkin Elmer luminescence spectrometer LS-55 (Waltham, MA, United States). The Ex/Em wavelengths used for single measurements were those described by Coble (1996): Peak-C (Ex/Em 340 nm/440 nm) as indicator of terrestrial-like substances, Peak-M (320 nm/410 nm) as indicator of marine-like substances, Peak-A (320 nm/410 nm) as indicator of generic humic-like substances, and Peak-T (280 nm/420 nm) as indicator of protein-like substances. HBA was determined by flow cytometry using a Becton Dickinson FACScalibur flow cytometer (Becton Dickinson, Franklin Lakes, NJ, United States) with a laser emitting at 488 nm (Gasol and del Giorgio, 2000). Subsamples of 400 μl were stained for 15 min with a SybrGreen deoxyribonucleic acid fluorochrome and then run at a flow rate of ca. 30 μl min^-1^. HBP was measured using the [^3^H] leucine (leu) incorporation technique (Kirchman et al., 1985) with the modifications of Smith and Azam (1992). Conversion of leu incorporation to bacterial production was done assuming a 0.073% of leu in protein and a C:P of 0.86 (Simon and Azam, 1989), using an isotope dilution factor of 2 typical for oligotrophic water and bacterial cell volumes determined by flow cytometry. Specific (sp) HBP was calculated dividing bulk HBP by the bacterial abundance.

The activities of three extracellular enzymes were determined using the fluorogenic substrates (all from Sigma–Aldrich Co. Ltd., MERCK, Darmstadt, Germany): L-Leu-7-amido-4-methyl-coumarin, 4-methylumbelliferyl-phosphate, and 4-methylumbelliferyl-β-D-glucoside for the enzymes leu-aminopeptidase (AMA), APA, and ß-glucosidase (ß-Gl), respectively. Subsamples (315 μl) and substrates (final concentration 125 μM) were added to black 96 microtiter-well-plates in four replicates. Fluorescence was measured at *t*_0_, immediately after addition of the substrate, and after incubations of approximately 15 min, 1 h, 3 h, and 5 h in an Infinite M200 Spectrofluorometer (Tecan, Männedorf, Switzerland) or a Modulus Microplate Reader (Turner BioSystems, Sunnyvale, CA, United States) at 365 nm Em and 450 nm Ex wavelengths. Incubations were performed inside the temperature-controlled chamber. The increase of fluorescence units during the incubation was converted into activity by preparing a standard curve with the end product of the reactions 7-amino-4-methyl-coumarin for AMA, and 4-methylumbelliferone for APA and β-Gl. Sp AMA and β-Gl, two enzymes synthesized mainly by heterotrophic bacteria (Sala et al., 2001, 2016), were calculated dividing bulk AMA and β-Gl by the bacterial abundance.

### Statistical Analysis

We evaluated the effect of aerosols on nutrient dynamics by subtracting their concentration at the sampling time before the addition to that at the sampling time after the addition (approximately 3 h later). Then nonparametric Wilcoxon tests (Wilcoxon, 1945) were conducted to look for differences between treatments (C, A, S). We also carried out Wilcoxon tests to evaluate the response of the biogeochemical variables to different conditions during the incubation period. Data were normalized to initial experimental values. We tested the experiments with the same characteristics: (1) the effect of the TREATMENT (C, A, S) was assessed considering all (*ALL* hereafter) the experiments but the WI one (as there were no S samples in this experiment); (2) differences between LOCATION (BCN, BLA, and OFF) were tested for the three SU experiments; (3) differences between SEASON (WI, SP, and SU) were tested in BCN; (4) differences between TREATMENT and the FILTRATION (samples filtered [F] by 0.8 μm with respect to those not filtered [NF]) were tested in the SU experiment in BCN (*SUBCN*). F samples were only considered for this particular analysis.

To analyze the correlation between the chemical and the biological response, we calculated the increase/decrease of a given variable in the aerosol-amended microcosms (A, S) with respect to C through the aerosol-induced ratio (AIR; Eq. 1) (Marín et al., 2017).

(1) $$\begin{array}{c} {\mathrm{AIR}}_{X(A,\;S)}=\frac{\frac{X_{F}}{X_{I}}(A,\;S)}{\frac{X_{F}}{X_{I}}(C)}, \end{array}$$

where *X_F_* is the maximum value reached for a given biological variable in a given time after the addition and *X_I_* is the value of the given variable before the addition. For nutrients, *X_F_* is the concentration at the sampling time just after the addition. An AIR value equal to 1 indicates that there is neither increase nor decrease for a given variable in the aerosol-amended microcosms (A, S) with respect to the C, whereas an AIR above or below 1 points to an increase or decrease of the variable in the amended containers, respectively. We then calculated the nonparametric Spearman’s ρ correlation coefficient for each pair of response variables. Three separate tests were performed: once for AIR values in the A samples (all data except the F samples), another for the S samples (all data except the F samples), and a specific test for the *SUBCN* samples, including all samples from this experiment (both treatments, F and NF). Significance was considered for *p* < 0.05. Analyses were performed with JMP (SAS) version 10.0 and R (v 3.2.4).

## Results

### Initial Characteristics of Surface Waters

The initial biogeochemical conditions of the seawater were different for the six experiments (**Table 1**). Inorganic nutrients were overall at higher concentration in BCN than in the other locations, the lowest concentrations found in BLA during SU and in the OFF station. In BCN, ammonium (${\mathrm{NH}}_{4}^{+}$${\mathrm{NH}}_{4}^{+}$) and total inorganic phosphorous (TIP) showed the highest concentrations during SP. In contrast, nitrate (${\mathrm{NO}}_{3}^{–}$), ${\mathrm{NH}}_{4}^{+}$, and TIP at the beginning of the WI experiment were at lower concentrations than expected at that time of the year, what we attribute to a post-bloom situation, since chlorophyll (Chl) concentration was quite higher than expected (Marín et al., 2017). The ratio between total inorganic nitrogen (TIN; as the sum of nitrate, nitrite, and ammonium) and TIP was close or lower to the Redfield ratio of 16 in all the experiments, except in BCN during WI and SU. TOC:TIP was always above the Redfield ratio of 106, but it was lowest in the SP experiments of BCN and BLA, and highest in SU. TOP and the biological variables were initially the lowest at the offshore station.

**Table 1:** Nutrient data (μmol l^-1^), chlorophyll concentration (Chl; μg l^-1^), heterotrophic bacterial abundance (HBA; cell ml^-1^), heterotrophic bacterial production (HBP; μg C l^-1^ d^-1^), and enzymatic activities (nmol l^-1^ h^-1^) measured at the beginning of the six experiments.

	Barcelona			Blanes		Offshore
	WI	SP	SU	SP	SU	SU
${\mathrm{NO}}_{3}^{–}$	1.32	1.71	1.82	1.63	0.51	0.34
${\mathrm{NH}}_{4}^{+}$	0.57	1.85	0.35	0.32	0.20	0.16
TIN	2.26	4.39	2.28	2.16	0.77	0.54
TIP	0.05	0.26	0.05	0.17	0.04	0.04
TOC	77	89	88	68	85	82
TOP	0.33	0.23	0.18	0.12	0.43	0.09
TIN:TIP	45	17	46	13	18	13
TOC:TIP	1546	341	1768	402	1973	1992
Chl	1.90	1.22	0.44	0.26	0.27	0.06
HBA(×10^5^)	5.06	11.51	6.6	4.70	8.03	4.57
HBP	4.53	1.19	1.91	3.51	3.79	0.08
AMA	26.93	49.64	10.45	9.21	39.34	0.28
β-Gl	0.12	1.10	1.20	0.15	2.91	0.12
APA	6.07	45.14	55.89	10.12	31.07	5.26

*AMA, leu-aminopeptidase; APA, alkaline phosphatase; ß-Gl: ß-glucosidase; WI = winter; SP = spring; SU = summer.*

### Aerosol Compositions and Their Effect on Nutrient Dynamics

The main constituents of S filters were silica and oxides of aluminum, while OC was the main component of the A aerosols, followed by silica (**Table 2**). The contribution of iron to TSP was higher in the S dust than in the A aerosols, while ${\mathrm{NO}}_{3}^{–}$, ${\mathrm{NH}}_{4}^{+}$, and P accounted for a higher percentage of TSP in the A than in the S filters.

**Table 2:** Chemical composition of the A (*N* = 16) and S (*N* = 6) filters used through the six experiments, as average and standard deviation.

	Anthropogenic (A)	Saharan (S)
TSP (μg m^-3^)	57.42 ± 44.88	137.62 ± 51.98
SiO_2_ (%TSP)	12.15 ± 10.72	30.02 ± 11.10
Al_2_O_3_ (%TSP)	4.05 ± 3.57	10.00 ± 3.70
Fe (%TSP)	2.25 ± 1.25	3.24 ± 0.99
TOC (%TSP)	18.79 ± 12.44	6.23 ± 1.64
${\mathrm{NO}}_{3}^{–}$ (%TSP)	6.74 ± 3.91	3.76 ± 1.41
${\mathrm{NH}}_{4}^{+}$ (%TSP)	1.16 ± 1.03	0.41 ± 0.17
P (%TSP)	0.12 ± 0.07	0.09 ± 0.03

*Data of major constituents are presented as percentage of TSP (total suspended particles).*

**FIGURE 2:**
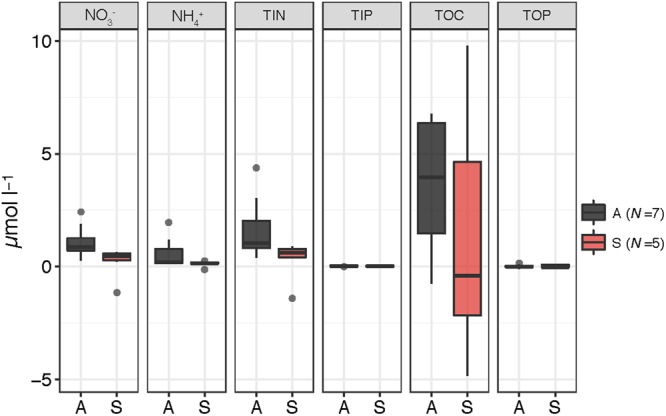
Box-plots showing the variation in the concentration (μmol l^-1^) of ${\mathrm{NO}}_{3}^{–}$, ${\mathrm{NH}}_{4}^{+}$, total inorganic nitrogen (TIN), total inorganic phosphorous (TIP), total organic phosphorous (TOP), and total organic carbon (TOC) after the aerosol additions – with respect to the values approximately 3 h before the addition – inside the A (*N* = 7) and S (*N* = 5) microcosms. The boxes indicate median and quartile values. The whiskers extend to 1.5 times the interquartile range. Values above or below this boundary are displayed as outliers.

After the aerosol additions, most nutrients increased their concentration in the amended microcosms (A, S), as expected, although the variability between experiments was high (**Figure 2**). TOC concentration decreased in the S microcosms in some of the experiments. Independent Wilcoxon tests (*N* = 19) showed that, after the amendment, ${\mathrm{NH}}_{4}^{+}$ increased significantly more in the A than in the C microcosms (*p* = 0.0043), and the increase in ${\mathrm{NO}}_{3}^{–}$ was statistically higher in the A than in both the C and the S containers (*p* = 0.0043 and *p* = 0.0118, respectively). TIN also increased significantly more in the A than in the C (*p* = 0.0043) and the S (*p* = 0.0454) microcosms. TIP and TOP only showed a moderate increase in the A and the S microcosms compared to the C, on average. Regarding the addition of dissolved organic compounds, FDOM measurements showed an increase in humic-like and protein-like substances after the aerosol additions in the amended microcosms in all the experiments, except in the filtered samples of BCN (**Supplementary Figure S1** in the Supplementary Information).

Considering the whole incubation period, ${\mathrm{NO}}_{3}^{–}$ and TIN were also significantly higher in the A containers than in the S and the C, and in the S than in the C (**Table 3**). TOC was statistically higher in the A than in both the S and the C microcosms. ${\mathrm{NH}}_{4}^{+}$ and TIP were significantly higher in the A than in the C. Considering the SU experiment of BCN in particular (*SUBCN)*, TIP was statistically higher in both amended microcosms than in the C. No significant differences between treatments were found for TOP in any of the comparisons tested.

**Table 3:** Results of the Wilcoxon test for the different comparison assessed. ‘Sp’ refers to specific rates of HBP, AMA, and β-Gl, respectively.

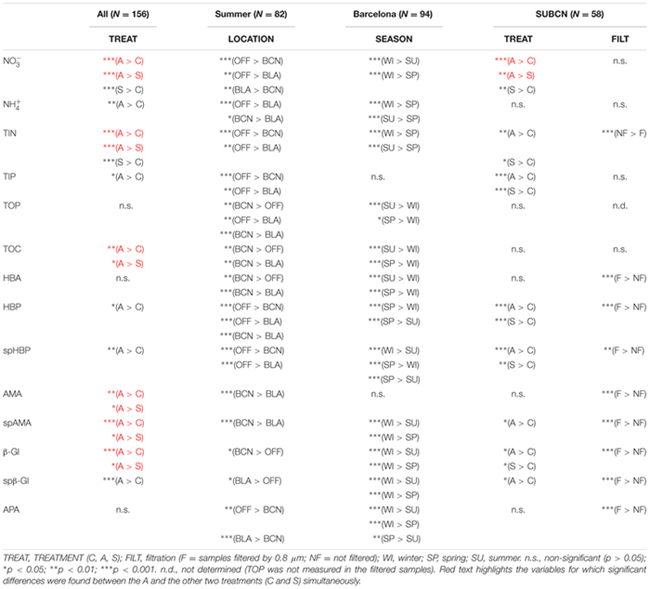

### Effect of Aerosols on Bacteria

**FIGURE 3:**
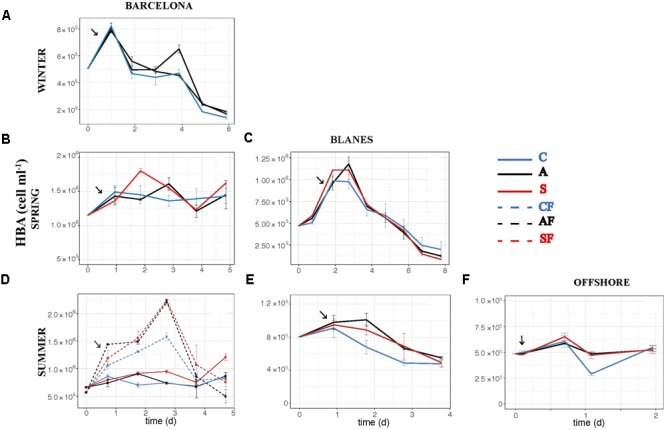
Heterotrophic bacterial abundance – HBA (cell ml^-1^) – over the incubation time in the six experiments: **(A)** WI-BCN, **(B)** SP-BCN, **(C)** SP-BLA, **(D)** SU-BCN, **(E)** SU-BLA, **(F)** SU-OFF. Error bars represent the standard error from two replicate containers. Arrows point to the moment of aerosol additions. C, controls; A, anthropogenic; S, Saharan; CF, controls filtered by 0.8 μm; AF, anthropogenic filtered by 0.8 μm; SF, Saharan filtered by 0.8 μm. Note that in the winter experiment there are two A treatments.

**FIGURE 4:**
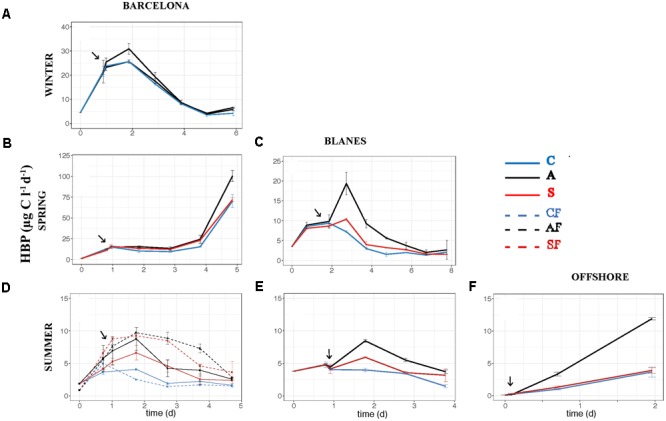
Heterotrophic bacterial production – HBP (μg C l^-1^ d^-1^) – over the incubation time in the six experiments: **(A)** WI-BCN, **(B)** SP-BCN, **(C)** SP-BLA, **(D)** SU-BCN, **(E)** SU-BLA, **(F)** SU-OFF. The same abbreviations and comments than in **Figure 3** are applicable.

**FIGURE 5:**
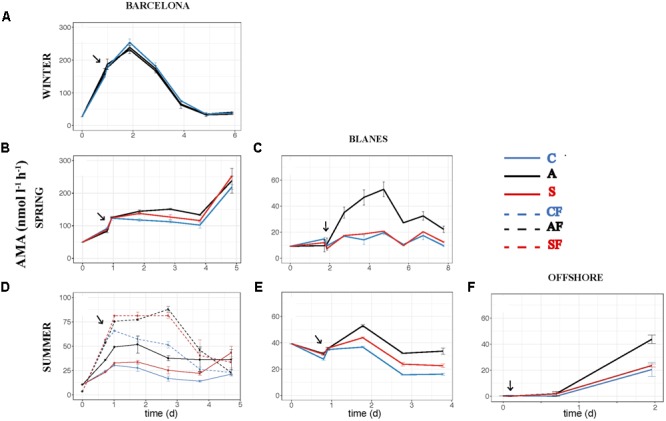
Activity of leu-aminopeptidase – AMA (nmol l^-1^ h^-1^) – over the incubation time in the six experiments: **(A)** WI-BCN, **(B)** SP-BCN, **(C)** SP-BLA, **(D)** SU-BCN, **(E)** SU-BLA, **(F)** SU-OFF. The same abbreviations and comments than in **Figure 3** are applicable.

**FIGURE 6:**
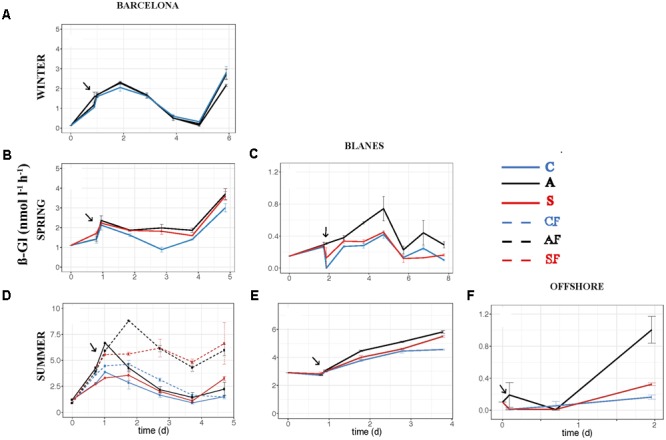
Activity of ß-glucosidase – ß-Gl (nmol l^-1^ h^-1^) – over the incubation time in the six experiments: **(A)** WI-BCN, **(B)** SP-BCN, **(C)** SP-BLA, **(D)** SU-BCN, **(E)** SU-BLA, **(F)** SU-OFF. The same abbreviations and comments than in **Figure 3** are applicable.

**FIGURE 7:**
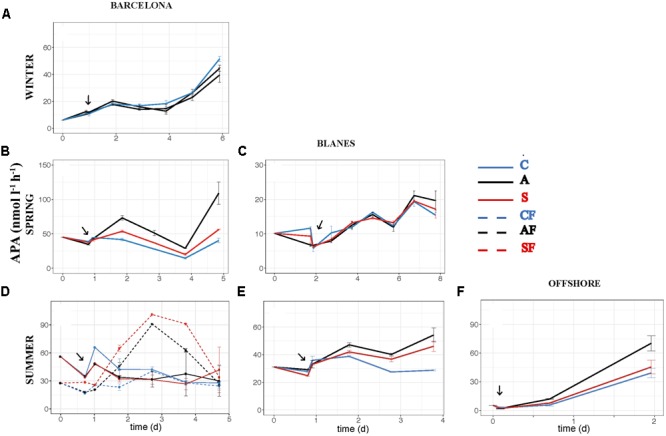
Activity of alkaline phosphatase – APA (nmol l^-1^ h^-1^) – over the incubation time in the six experiments: **(A)** WI-BCN, **(B)** SP-BCN, **(C)** SP-BLA, **(D)** SU-BCN, **(E)** SU-BLA, **(F)** SU-OFF. The same abbreviations and comments than in **Figure 3** are applicable.

The effect of atmospheric particles on bacterial abundance was moderate and dependent on the location, with AIR values always very close to 1 (**Supplementary Table S1**). Bacterial abundance was more stimulated with S dust in BCN and offshore, whereas the response was higher with A aerosols in BLA (**Figure 3**). Average AIR values for HBA (without considering the F samples) were 1.1 ± 0.2 in both the A (*N* = 14) and the S (*N* = 10) samples. Instead, AIR values for bacterial production and extracellular enzymatic activities were usually larger than 1 in all the experiments but in WI (**Supplementary Table S1**), pointing to a positive effect of aerosols. The temporal evolution of these variables shows that the enhancement was overall higher in the A samples (**Figures 4–7**). AIR values ranged from 0.9 to 3.3 for HBP, from 0.8 to 7.1 for AMA, from 0.5 to 7.1 for β-Gl, and from 0.7 to 3.2 for APA in the A samples (*N* = 14). S AIR values ranged from 1 to 2.1 for HBP, from 1 to 1.7 for AMA, from 0.6 to 2.1 for β-Gl, and from 0.5 to 1.6 for APA (*N* = 10). On average, AIR values for HBP and enzymatic activities (*N* = 4) were 1.4 times larger in the A microcosms than in the S. As the effect on bacterial abundance was overall low, sp production (spHBP) and enzymatic activities (spAMA and spβ-Gl) were still higher in the A than in the other microcosms. More specifically, AIR values were 1.2 times higher in the A containers than in the S (*N* = 3). AIR values ranged from 0.8 to 4.3 for spHBP, from 0.6 to 14.6 for spAMA, and from 0.6 to 7.5 for spβ-Gl in the A samples (*N* = 14). In the S samples (*N* = 10), AIR values ranged from 0.8 to 2.4 for spHBP, from 1 to 6 for spAMA, and from 0.8 to 3.2 for spβ-Gl. On the other hand, in BCN, in the SU experiment (*SUBCN*), all the biological variables showed a high response in the A and S filtered samples (AF and SF) compared with the filtered controls (CF) (**Figures 3D, 4D, 5D, 6D, 7D**). AIR values averaged 1.03 ± 0.03 for HBA, 3.39 ± 0.11 for HBP, 1.44 ± 0.07 for AMA, 1.49 ± 0.33 for β-Gl, 1.97 ± 0.55 for APA, 4.76 ± 0.38 for spHBP, 1.61 ± 0.05 for spAMA, and 3.23 ± 0.42 for spβ-Gl in the AF samples (*N* = 2). In the SF samples (*N* = 2), average AIR values were 1.28 ± 0.22, 3.02 ± 0.77, 1.31 ± 0.00, 1.40 ± 0.20, 1.43 ± 0.29, 2.80 ± 0.18, 1.09 ± 0.18, and 1.91 ± 0.55 for HBA, HBP, AMA, β-Gl, APA, spHBP, spAMA, and β-Gl.

#### Effect of Aerosols at Different Locations and Times of the Year

The effect of atmospheric particles on seawater was statistically assessed comparing the results from all the experiments except the WI one (*ALL*) (**Table 3**). Results show that AMA, spAMA, and β-Gl were significantly higher in the A than in the other microcosms, and HBP, spHBP, and spβ-Gl were statistically higher in the A than in the C. Comparing the effect of both aerosols at different locations, we found that HBP and spHBP were significantly more stimulated offshore than in BCN and BLA. APA was higher offshore and in BLA than in BCN. HBA, AMA, spAMA, and ß-Gl were statistically higher in BCN, and spß-Gl was higher in BLA than OFF. Inorganic nutrients were overall significantly higher offshore than in the coastal locations, while TOP and TOC were statistically higher in BCN. Regarding the effect of the season – assessed for the three experiments in BCN – HBA and HBP were statistically larger in SP and SU than in WI, while enzymatic activities were higher in WI than in the other seasons. Inorganic N was significantly higher in WI than in the other seasons, whereas TOP and TOC were larger in SU and SP than in WI.

**FIGURE 8:**
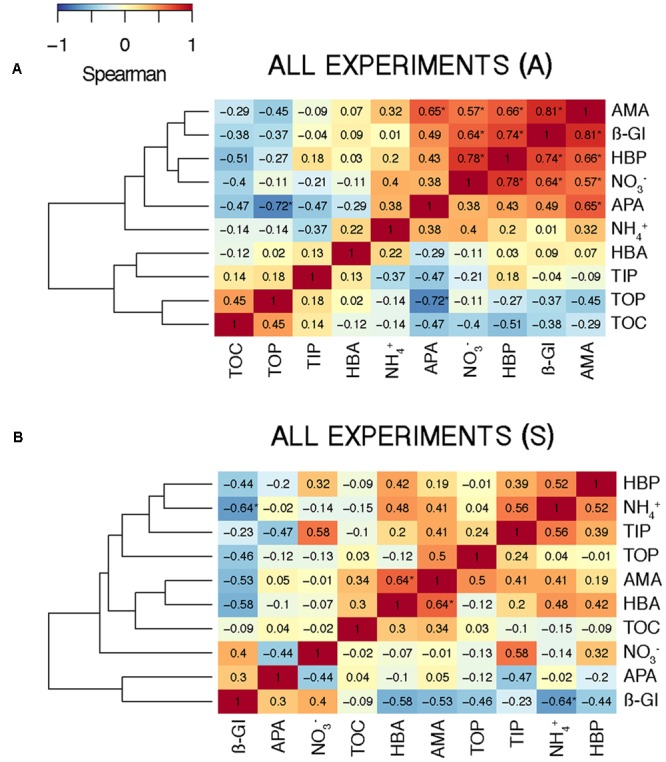
Heat map showing the Spearman’s ρ coefficient between the biogeochemical variables considering **(A)** the A (*N* = 14) and **(B)** the S (*N* = 10) samples from all the experiments. Significant correlations (*p* < 0.05) are indicated with an asterisk (^∗^).

To investigate the overall relationship between the elements released by the aerosols and the response in the biological variables – calculated as the AIR value (section “Statistical Analysis”) – we calculated the Spearman’s ρ coefficient for each pair of variables in the A and the S microcosms. In the A samples, the biological variables, except HBA, showed a high positive correlation among them and with ${\mathrm{NO}}_{3}^{–}$ (**Figure 8A**). The correlation with ${\mathrm{NO}}_{3}^{–}$ was significant for HBP, AMA, and β-Gl. In the S samples, correlations were moderate (**Figure 8B**). In this case, the biological variables showed a higher correlation with TIP than with ${\mathrm{NO}}_{3}^{–}$. The only positive correlation that was significant was that between HBA and AMA.

#### Direct Effect of Aerosols on Bacteria

**FIGURE 9:**
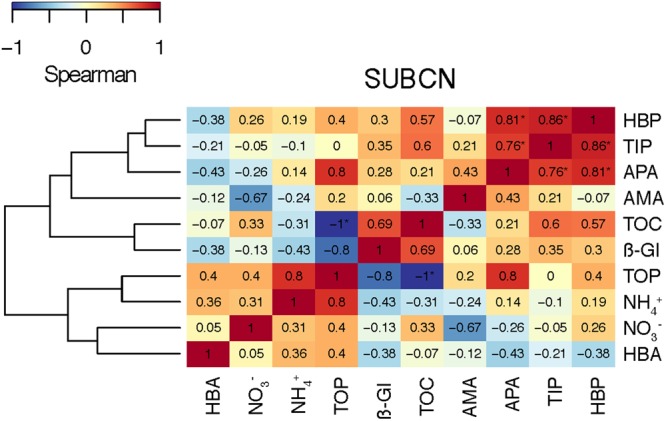
Heat map showing the Spearman’s ρ coefficient between the biogeochemical variables in the SU experiment of BCN (*SUBCN*). Samples from both aerosol treatments (A and S), and both, F and NF, have been included (*N* = 8). Significant correlations (*p* < 0.05) are indicated with an asterisk (^∗^).

In *SUBCN*, the effect of aerosols on bacterial abundance and activity was evaluated with and without competitors and predators, further filtering the seawater by 0.8 μm in 6 of the 15 l containers (section “Experimental Design and Analytical Procedures”). Statistical analyses showed that HBP, spHBP, and β-Gl were significantly higher in both amended microcosms than in the C, and spAMA and spβ-Gl were more stimulated in the A containers than in the C (**Table 3**). All the biological variables were significantly more enhanced in the containers filtered by 0.8 μm than in those unfiltered. Furthermore, HBA responded quicker to the aerosols in the filtered samples, showing a peak 2 days after the addition (**Figure 3D**). In this particular experiment, considering the F and NF samples altogether, most of the biological variables showed a high positive correlation with TIP, which was significant in the case of HBP and APA (**Figure 9**).

## Discussion

### Nutrients Released by Aerosols

Aerosol composition of both S and A filters was overall within the ranges reported in the literature for the NW Mediterranean (Viana et al., 2005; Pey et al., 2010; Escudero et al., 2011). Nutrient measurements inside the microcosms before and after the aerosol additions (time lag ≈ 3h) showed that S dust released on average 0.25 ± 1.01 μmol TIN mg^-1^, 0.02 ± 0.02 μmol TIP mg^-1^, and 1.67 ± 6.91 μmol TOC mg^-1^. These values are within the reported values of TIN dissolved from S dust in previous experiments carried out in the Mediterranean (Herut et al., 2005, 2016; Lekunberri et al., 2010). TIP concentration is higher than those usually found in the bibliography but within the range reported by Pulido-Villena et al. (2008) after a real deposition event of S dust (2.58 g m^-2^) in the NW Mediterranean (release of P: 0.03 μmol mg^-1^), and in microcosm experiments adding similar concentrations of dust than in our experiments (0.02–0.03 μmol mg^-1^). TOC showed high variability between experiments, especially in the S microcosms. It sometimes increased after adding the dust, but in other occasions it showed no increase or even decrease (**Figure 2**). TOC concentrations in surface waters of the Catalan coast range from 50 to ca. 150 μmol l^-1^ (Vila-Reixach et al., 2012; Romera-Castillo et al., 2013; **Table 1**). Thus, the amount of TOC supplied by aerosols is usually much lower than the actual concentration in the seawater. In situations of OC co-limitation, it may be quickly taken up by heterotrophic bacteria, making difficult the determination of TOC released by aerosols. Although there was no apparent OC co-limitation at the beginning of our experiments, the increase in inorganic nutrients after the aerosol additions may have produced an unbalance in the Redfield ratio in the microcosms, yielding to the quick consumption of TOC. Alternatively, the decrease in TOC determined in some experiments may be associated to the error of the analytical procedure, that is in the same range of the TOC concentration released by the aerosols in the case of the S samples (CV = 1.44 ± 0.76%). A high variability in TOC supplied by aerosols has also been reported in the literature (Duarte et al., 2006; Pulido-Villena et al., 2008; Lekunberri et al., 2010).

Anthropogenic particles released higher amounts of TIN (1.98 ± 1.79 μmol TIN mg^-1^) and TOC (4.70 ± 3.45 μmol TIN mg^-1^), but the same concentration of TIP per milligram of aerosol than S dust. TIN released by these aerosols was slightly higher than that reported by Guo et al. (2014) (1 μmol mg^-1^), but much lower than that determined by Bonnet et al. (2005) and Duarte et al. (2006) (∼24 μmol mg^-1^). The similar concentration of P released from both types of particles, higher than expected for S particles, must be related to the fact that S particles arriving to the NE of the Iberian Peninsula are mixed with local and regional sources of pollution during their trajectory. Pollution involves increases in NOx and SOx compounds, the main acid precursors species in the atmosphere (Stockdale et al., 2016), and the solubility of P increases with acidity (Nenes et al., 2011; Stockdale et al., 2016). Hence, it seems reasonable to find a higher release of P from dust collected in the Catalan coast than in other experiments performed close to the desert or in more remote areas less exposed to pollution (e.g., Bonnet et al., 2005; Herut et al., 2005; Guo et al., 2016). In fact, Stockdale et al. (2016) suggested that the flux of bioavailable P from acidified dust is expected to increase in the Mediterranean Sea during the coming years.

The additions of TOC released with both aerosols (from 0 to 7 μmol l^-1^) are negligible compared to ambient concentration, as pointed above. However, P released by the atmospheric particles (0.02 ± 0.02 μmol l^-1^) is in the same range of P annual concentration in the offshore station (Lazzari et al., 2016), and within the range of concentrations observed during the summer stratification period in BCN (Arin et al., 2013; Romero et al., 2014) and BLA (Guadayol et al., 2009) coastal waters. Thus, both aerosols have the potential to be an important source of P during the stratification period in coastal waters, and during the whole year in offshore waters of the NW Mediterranean Sea. TIN released by the S dust was overall small compared to ambient concentrations in western Mediterranean coastal waters, but it might also be an important source of inorganic N during the stratification period, when TIN concentration in the seawater can be <0.1 μM (Guadayol et al., 2009; Arin et al., 2013; Romero et al., 2014). The average TIN concentration leached by the A particles (1.67 μmol l^-1^) is equivalent to the annual average concentration in BLA and in the offshore station, and about three times higher than that during the stratification period (Gasol et al., 2012; Lazzari et al., 2016). This concentration is also close to the TIN summer average in BCN (Arin et al., 2013; Romero et al., 2014). A aerosols have therefore the potential to constitute an important source of bioavailable N during the whole year in coastal locations of the NW Mediterranean, but especially during the stratification period.

### Aerosol Effect on Bacterial Abundance and Metabolism

Our results show that HBP was significantly enhanced with both types of atmospheric particles (in the SU experiment of BCN), or only with the A particles (when all the experiments were considered together), whereas aerosols did not yield significant increases in bacterial abundance overall. In a previous work where only results from the BCN experiments were considered, we found a significantly higher enhancement of bacterial abundance with S dust (Marín et al., 2017). This evidences the importance to perform several experiments, considering different initial conditions, to be able to draw general patterns regarding the effect of aerosols in marine surface waters, especially in the more variable coastal environment. In other previous studies carried out with S dust, a great stimulation of bacterial production was observed, in comparison with no, or lower, response on bacterial abundance (e.g., Duarte et al., 2006; Lekunberri et al., 2010; Marañón et al., 2010; Pulido-Villena et al., 2014). Studies that evaluated the effect of A aerosols on bacterial abundance did not observe significant increases either (Bonnet et al., 2005; Ternon et al., 2011), while Herut et al. (2016) noticed a larger increase in bacterial production than in bacterial abundance after the addition of A aerosols in eastern Mediterranean surface waters. Teira et al. (2013) found a higher effect on bacterial production than on abundance in experiments with rainwater added to coastal eutrophic waters of the Ría de Vigo (NW Spain). We did observe a significant increase of HBA in the filtered samples compared to those not filtered, though (**Figure 3D** and **Table 3**), pointing to the importance of competition and grazing processes controlling bacterial growth and masking effects on biomass. We discuss the results from this particular experiment further below.

We also looked at the activity of three extracellular enzymatic activities: AMA, APA, and β-Gl. Extracellular enzymes can be synthetized by several organisms, including bacteria, phytoplankton, heterotrophic nanoflagellates, and zooplankton (Karner et al., 1994; Jamet and Boge, 1998). Nonetheless, APA is mainly synthesized by phytoplankton, whereas AMA and β-Gl are mainly produced by bacteria (Vives-Rego et al., 1985; Münster et al., 1992). Thus, the enzymatic activities related with bacteria were the ones more stimulated with A aerosols (AMA, spAMA, β-Gl, and spβ-Gl). An increase in AMA activity is indicative of either TIN limitation or a supply of dissolved organic N, while an increase in β-Gl points to a higher hydrolysis of carbohydrates, mainly derived from cellulose (Rath et al., 1993; Kamer and Rassoulzadegan, 1995; Sala et al., 2001, 2016; Misic et al., 2008). As A particles produced a significant increase in inorganic N (and in the N:P ratio), we can discard the stimulation of AMA as indicative of TIN limitation. Hence, the observed enhancement of AMA and β-Gl activities determined in these experiments in the A microcosms must be attributed to either an addition of organic sources of N and C, or the hydrolysis of polymeric substrates already present in the seawater that can be mineralized thanks to the supply of inorganic nutrients that could be limiting bacterial metabolic processes.

In aerosol-amended experiments where the whole planktonic community is assessed, bacteria might take up the organic compounds needed for growth either from the atmospheric particles or from those released by phytoplankton cells. The concomitant increase of HBP and the three enzymatic activities observed toward the end of the experiment carried out in BCN in SP (**Figures 4B, 5B, 6B, 7B**) may indicate a release of organic compounds from phytoplankton cells, as we observed a peak of Chl 1 day after the aerosol additions, and then phytoplankton concentration quickly decreased toward the end of the experiment, and so decreased TIN and TIP (Marín et al., 2017). However, the enhancement of HBP and enzymatic activities observed in BLA during SP and in the three SU experiments is unlikely an indirect effect from phytoplankton released compounds, since HBP and enzymatic activities started to increase earlier or at the same time as Chl in these experiments (data not shown). Aerosols, especially of A origin, are also a source of OC (Viana et al., 2005; Pateraki et al., 2012), as reflected in the increase in humic-like and protein-like substances (**Supplementary Figure S1**). However, these compounds may be of recalcitrant nature, as bacteria did not take them up through the incubation time (Sánchez-Pérez et al., 2016). More specifically, the enhancement of β-Gl suggests that A particles may be a source of carbohydrates. The main source of these compounds to the atmosphere is biomass burning (Pöschl, 2005), probably coming from local waste incineration. The stimulation of AMA also suggests a supply of organic N. Thus, although, we did not find a correlation between the TOC released by the aerosols and the biological response (**Figures 8, 9**), the stimulation of AMA and β-Gl points to the exhaustion of the labile fraction of organic compounds in the seawater (Chróst, 1990) and the hydrolysis of polymeric substrates that either were already present or came from the aerosols.

Regarding the inorganic nutrients, we found that A aerosols released significantly more nitrate and TIN than S dust. Hence, the higher stimulation of HBP and enzymatic activities observed in the A samples points to inorganic N (and more specifically nitrate) as the major driver in the observed response. In fact, considering all the results from the six experiments, most of the biological variables showed a high correlation with nitrate in the A samples (**Figure 8A**). The biological response to S dust instead was not significant when considering all the experiments, but the SU experiment of BCN (*SUBCN*) (**Table 3**). Interestingly, considering both the A and S samples in *SUBCN*, we found a significant positive correlation between TIP, HBP, and APA, and it was also higher between TIP and AMA and β-Gl, than between ${\mathrm{NO}}_{3}^{–}$ and these biological variables (**Figure 9**). One could expect an inverse correlation between APA and TIP (Sala et al., 2001, and references therein). However, the positive correlation between TIP and APA found in this experiment can be explained because the AIR value for TIP was calculated considering the moment just after the aerosol additions (section “Statistical Analysis”), while maxima APA values were found toward the end of the experiment (**Figure 7D**), when TIP was starting to decrease. In this experiment, aerosols released a higher concentration of phosphate than in other experiments, and the concentration of N related to that of P at the beginning of the *SUBCN* experiment (N:P ratio = 46) was much larger (**Table 1**), being P the main limiting nutrient in this case. In all, the increase observed in HBP and enzymatic activities with the atmospheric – especially the A – particles should be a combination of two different factors: (1) the release of inorganic nutrients (i.e., TIN and TIP) and some organic compounds from the aerosols, and (2) the re-mineralization of particulate organic matter already present in the seawater. The final impact will be determined by the chemical composition of the aerosols and the initial status of the seawater. In agreement with our results, Herut et al. (2016) pointed to N as the main driver of the higher response observed in the microbial community with A particles compared to S dust in the eastern Mediterranean, whereas Pitta et al. (2017) pointed to both N and P as the main components released by dust triggering a response in the bacterial community when seawater was N and P co-limited (N:P = 10). Therefore, as A aerosols release more inorganic N than P, these particles have a huge potential to stimulate bacterial activity when both N and P are in low concentrations, a common situation in coastal (Guadayol et al., 2009) and open waters (Moutin et al., 2012) of the western Mediterranean during the stratification period. N has been considered the main limiting nutrient in most of the oligotrophic ocean (e.g., Moore et al., 2013). However, N_2_ fixation may be important in some of these areas (Bonnet et al., 2011), where phosphorous and iron could become the main limiting nutrients. In any case, as oligotrophic marine areas represent more than 50% of the global ocean (Antoine et al., 1996), it is important to address the effect of an increasingly polluted and acidified atmosphere (IPCC, 2014; Stockdale et al., 2016) in these regions.

When evaluating the effect of the location in SU, a larger stimulation of HBP, spHBP, and APA was observed offshore than in the coastal locations. Marañón et al. (2010) showed that the effect of aerosols on HBP increased with increasing oligotrophy. Hence, the larger increase of these variables observed offshore can be attributed to the higher oligotrophy of the open ocean waters offshore Balearic Islands (Chl annual average of 0.25 ± 0.09 μg l^-1^; Gallisai et al., 2014) in comparison with the coastal waters of BCN (Chl average of 1.58 ± 1.09 μg l^-1^; Romero et al., 2014) and BLA (Chl average of 0.63 ± 0.05 μg l^-1^; Guadayol et al., 2009). In fact, inorganic nutrients and Chl concentration were much lower at the beginning of the offshore experiment than in the others (**Table 1**), and inorganic N and P were significantly higher after the aerosols addition offshore than in the other experiments (**Table 3**). It must be noted that offshore waters were amended with aerosols from BCN, which are probably richer in N, P, and TOC than actual aerosols over the atmosphere of the OFF station. In contrast, HBA, AMA, spAMA, and β-Gl were more stimulated in BCN. TOC and TOP were significantly higher in BCN, but it is difficult to establish causality, as no clear correlation was observed between TOC and TOP and the biological variables, and both remained at similar concentrations in all microcosms through the incubation time. With respect to the season, HBA and HBP were more enhanced in SU and SP than in WI. In SU, this was attributed to the high oligotrophy of the initial water, with P being the main limiting nutrient, and aerosols leaching more P than in other experiments, as previously said. While in SP, although the initial water was rich in both N and P (**Table 1**), both started to decrease quickly, the extra nitrate added with the aerosols rapidly taken up by phytoplankton, yielding a subsequent increase in HBP (Marín et al., 2017). Enzymatic activity instead was higher in WI than in the other seasons, but without apparent differences between treatments (**Figures 5A, 6A, 7A**). In the WI experiment, an increase in Chl was observed the day after the addition in all the microcosms, followed by a decrease toward the end of the experiment (Marín et al., 2017). Thus, the major stimulation of enzymatic activities observed in WI in all the microcosms is likely due to the remineralization of organic compounds already present in the seawater.

Finally, we have shown that bacterial abundance and activity can be directly stimulated by atmospheric deposition, and that the effect is higher without the presence of competitors (i.e., phytoplankton) and predators (i.e., heterotrophic nanoflagellates). These results explain why previous studies where only bacteria were considered (e.g., Pulido-Villena et al., 2008) showed a higher effect of S dust on bacterial abundance with respect to other experiments with the whole planktonic community, at similar dust additions (e.g., Herut et al., 2005; Pulido-Villena et al., 2014). More experiments testing the effect of aerosols on the heterotrophic prokaryote community with and without the presence of competitors and predators need to be carried out to confirm our results, though. No significant differences were found in nutrient concentration between the filtered and not filtered samples, except, surprisingly, for TIN (**Table 3**). TIN tended to decrease toward the end of the experiment in all the samples, but one of the S containers showed a second peak on days 3 and 4, probably associated to the remineralization of organic matter already present in the seawater. Although this peak was of not enough intensity to represent a difference in the biological activity, much higher in the F samples, this might be the reason of the higher content in TIN found in the NF samples. It must also be pointed out that the effect of both types of aerosols in the F samples was similar for almost all the biological variables, except β-Gl and spβ-Gl, which were higher in the A microcosms (**Figures 3D, 4D, 5D, 6D, 7D**; *SUBCN*). These observations suggest that, in the absence of competitors and predators, bacteria show a similar preference to both types of aerosols. Bacterial losses later in the experiment may be attributed to virus or residual nanoflagellates passing through the 0.8 μm filter and grazing, unchecked themselves. In any case, these losses are also similar in both A and S. We did not found significant differences in the concentration of heterotrophic nanoflagellates in the unfiltered A and S samples in any of the experiments (data not shown). Hence, the higher bacterial production and activity of AMA observed with the A particles when the whole community was evaluated must then be attributed to either the indirect stimulation after phytoplankton growth with these particles (which may be the case of BCN during SP) or to the presence of other competitors for S dust. For instance, cyanobacteria were significantly more stimulated with S dust than with the other treatments in the experiment carried out in BLA during SU (Marín et al., in preparation).

## Conclusion

Considering data from six experiments carried out in the NW Mediterranean, we show that A particles enhance 1.4 times more bacterial production and enzymatic activity than S dust at the same particle mass. Significant differences between the A and the other treatments were found for HBP, spHBP, AMA and spAMA activities, and β-Gl and spβ-Gl activities. The larger stimulation with A particles is attributed to the major release of bioavailable inorganic (mainly ${\mathrm{NO}}_{3}^{–}$) compounds. The effect is variable depending on the initial status of the seawater, though. When initial waters were poor in inorganic nutrients (but rich in OC; i.e., in SU), bacteria outcompeted phytoplankton in responding to the inorganic nutrients released by aerosols, whereas at other times the effect on bacteria was mediated through a previous stimulation of phytoplankton. When no bacterial competitors or predators are present, both A and S atmospheric particles yield a major enhancement of bacterial abundance and activity.

## Author Contributions

IM, SN, and FP conceived and designed the study. IM, SN, EDS-P, ET, CA, MMS, CM, and FP collected and analyzed the samples, and all contributed to data interpretation. IM wrote first draft of the manuscript to which all authors contributed in subsequent revisions. All authors approved the final version.

## Conflict of Interest Statement

The authors declare that the research was conducted in the absence of any commercial or financial relationships that could be construed as a potential conflict of interest.

## Footnotes

1 www.calima.ws

## Supplementary Material

FIGURE S1 Changes in the excitation (Ex)–emission (Em) matrixes of FDOM after the aerosol addition in the six experiments. The value of florescence before the addition in all the microcosm – expressed in quinine sulfate units (QSU) – is the same than in the C, whereas A and S matrixes show the values after the additions. The different peaks are indicated: Peak-C (terrestrial-like substances), Peak-M (marine-like substances), Peak-A (generic humic-like substances), and Peak-T (280 nm/420 nm) (protein-like substances). In the WI experiment there were no Saharan microcosms but A particles with different composition (A1 and A2) were added to two duplicate microcosm each.

TABLE S1 Aerosol-induced ratios (AIR) of the biogeochemical variables studied (abbreviations as in the main text). TREAT, TREATMENT (A = anthropogenic, S = Saharan); FILT, filtration (F = samples filtered by 0.8 μm; NF = not filtered); WI, winter; SP, spring; SU, summer; BCN, Barcelona; BLA, Blanes; OFF, offshore. A dash indicates that the ratio could not be calculated.

## Abbreviations

A: anthropogenic
AMA: leucine-aminopeptidase
APA: alkaline phosphatase
BCN: Barcelona
ß-Gl: ß-glucosidase
BLA: Blanes
C: controls
F: samples filtered by 0.8 μm
FILT: filtration process
NF: samples not filtered by 0.8 μm
OFF: offshore location
S: Saharan
SP: spring
SU: summer
SUBCN: Summer-Barcelona
TREAT: experimental treatment
WI: winter
