# Supplementary figures and images for: Coastal Bacterioplankton Metabolism Is Stimulated Stronger by Anthropogenic Aerosols than Saharan Dust
Source: Front Microbiol. 2017 Nov 15;8:2215. doi: 10.3389/fmicb.2017.02215 (PMC5694759; doi:10.3389/fmicb.2017.02215)

WI-BCN

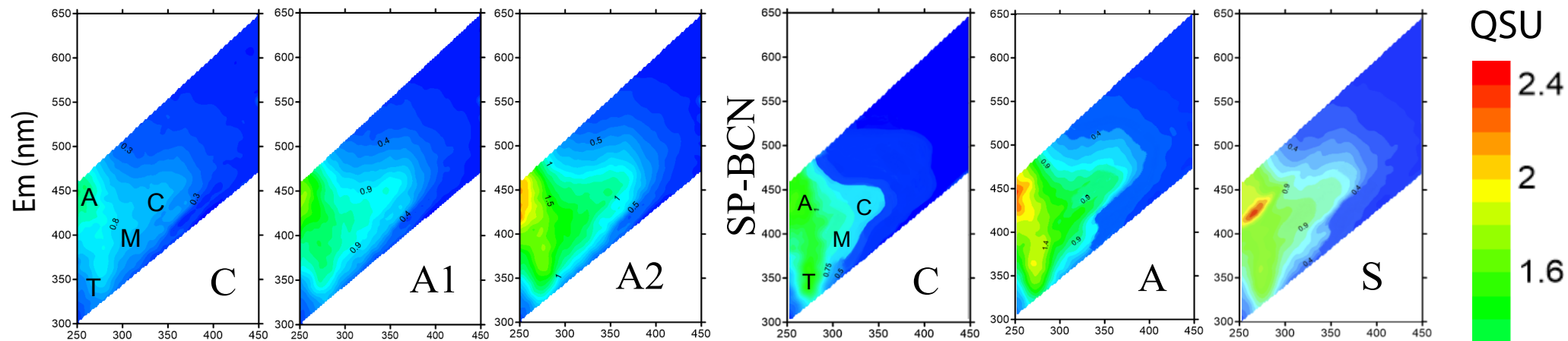

SU-BCN

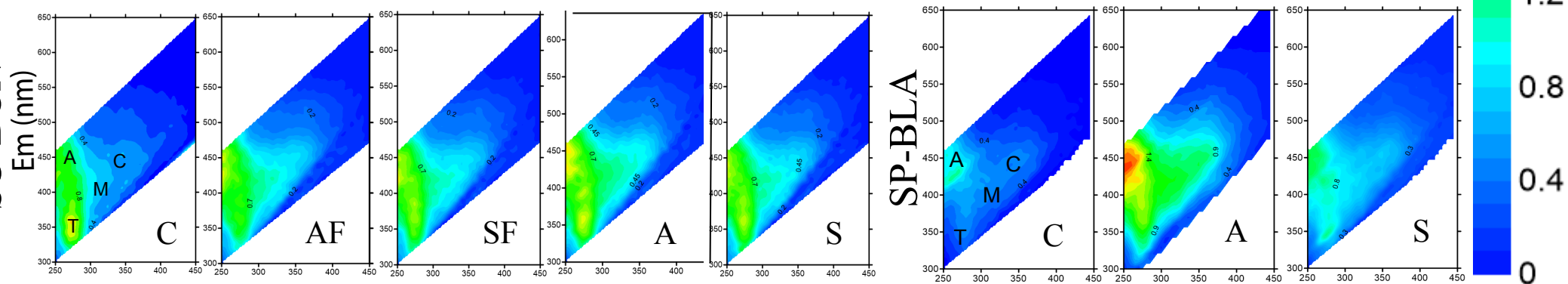

SU-BLA

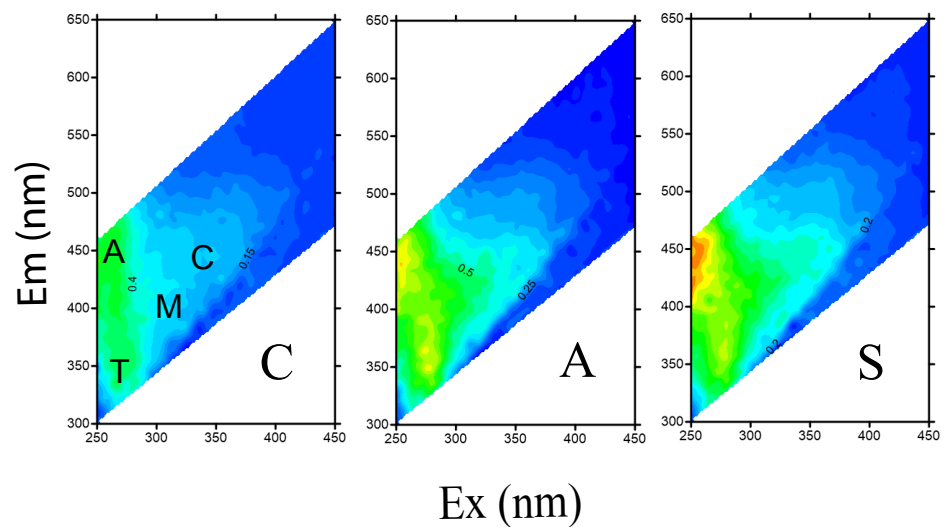

SU-OFF

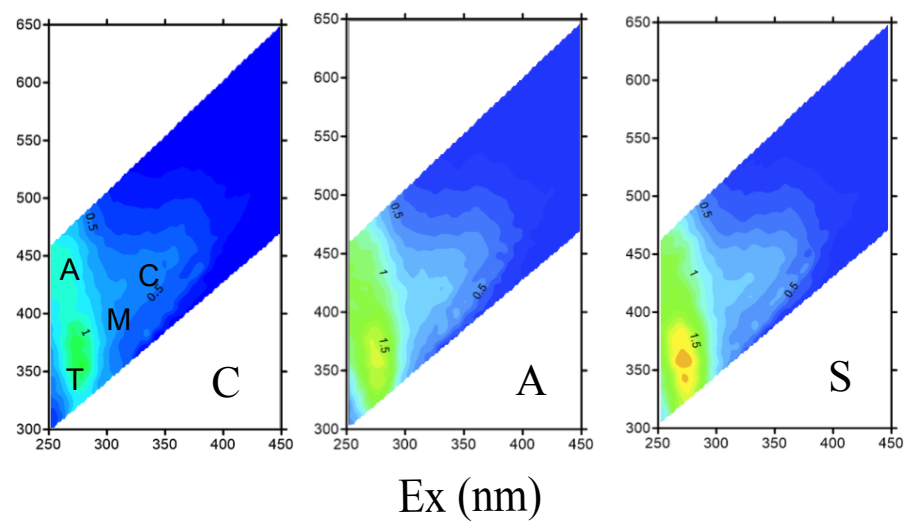

SP-BCN

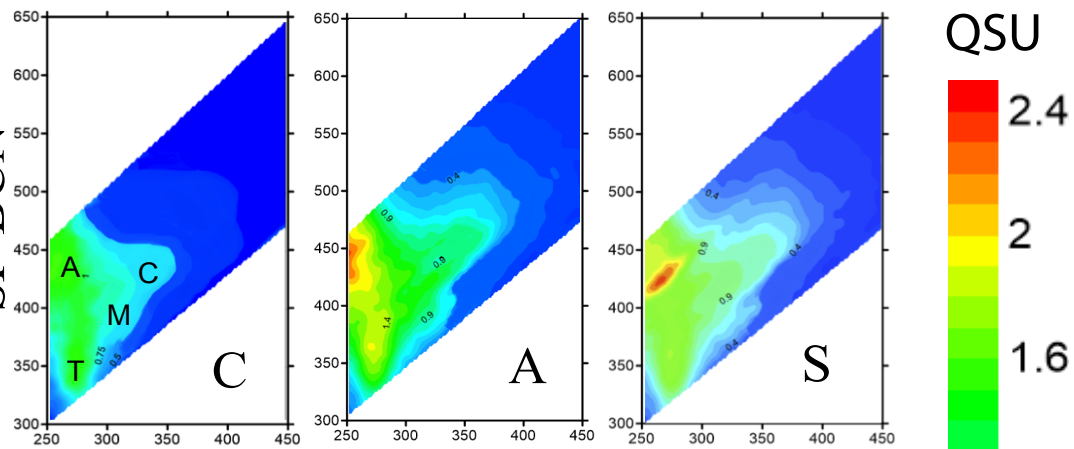

SP-BLA

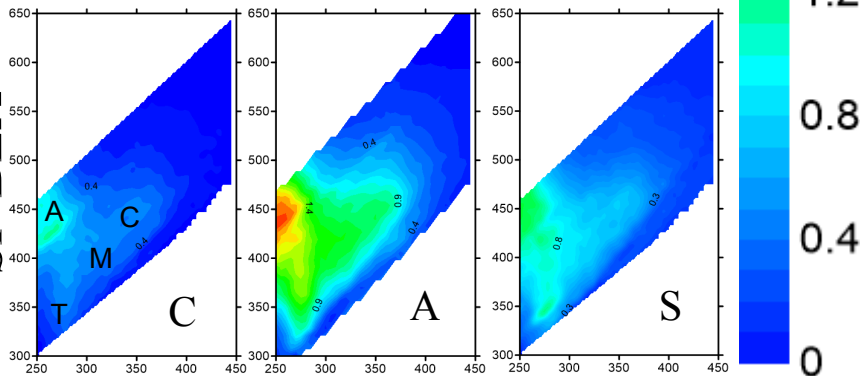

QSU

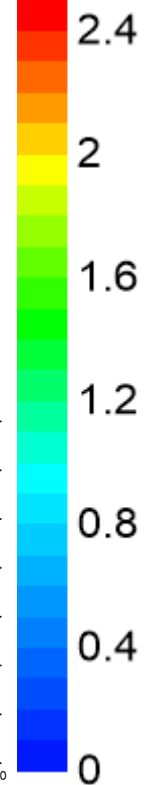

Supplement: FIGURE S1 — Changes in the excitation (Ex)–emission (Em) matrixes of FDOM after the aerosol addition in the six experiments. The value of florescence before the addition in all the microcosm – expressed in quinine sulfate units (QSU) – is the same than in the C, whereas A and S matrixes show the values after the additions. The different peaks are indicated: Peak-C (terrestrial-like substances), Peak-M (marine-like substances), Peak-A (generic humic-like substances), and Peak-T (280 nm/420 nm) (protein-like substances). In the WI experiment there were no Saharan microcosms but A particles with different composition (A1 and A2) were added to two duplicate microcosm each. [file Image_1.PDF]
