# Supplementary material for: Coastal Bacterioplankton Metabolism Is Stimulated Stronger by Anthropogenic Aerosols than Saharan Dust
Source: Front Microbiol. 2017 Nov 15;8:2215. doi: 10.3389/fmicb.2017.02215 (PMC5694759; doi:10.3389/fmicb.2017.02215)
Supplement: TABLE S1 — Aerosol-induced ratios (AIR) of the biogeochemical variables studied (abbreviations as in the main text). TREAT, TREATMENT (A = anthropogenic, S = Saharan); FILT, filtration (F = samples filtered by 0.8 μm; NF = not filtered); WI, winter; SP, spring; SU, summer; BCN, Barcelona; BLA, Blanes; OFF, offshore. A dash indicates that the ratio could not be calculated. [file Table_1.DOCX]

| **SEASON** | **LOCATION** | **TREAT** | **FILT** | **AIR-NO_3_** | **AIR-NH_4_** | **AIR-TIN** | **AIR-TIP** | **AIR-TOC** | **AIR-TOP** | **AIR-HBA** | **AIR-HBP** | **AIR-spHBP** | **AIR-AMA** | **AIR-spAMA** | **AIR-ß-Gl** | **AIR-spß-Gl** | **AIR-APA** |
| --- | --- | --- | --- | --- | --- | --- | --- | --- | --- | --- | --- | --- | --- | --- | --- | --- | --- |
| WI | BCN | A | NF | 1.40 | 1.89 | 1.38 | 1.27 | 1.14 | 5.04 | 1.00 | 1.34 | 0.96 | 0.84 | 0.78 | 0.54 | 0.69 | 0.77 |
| WI | BCN | A | NF | 1.32 | 1.34 | 1.26 | 1.26 | 1.13 | 11.03 | 0.99 | 0.99 | 0.83 | 0.85 | 0.63 | 0.76 | 0.59 | 0.68 |
| WI | BCN | A | NF | 2.53 | 4.37 | 2.71 | 0.95 | 1.19 | 3.38 | 1.00 | 0.94 | 0.90 | 0.98 | 0.90 | 0.85 | 0.66 | 0.97 |
| WI | BCN | A | NF | 2.44 | 4.44 | 2.63 | 1.04 | 1.05 | 2.30 | 1.00 | 0.96 | 0.97 | 0.88 | 0.86 | 1.14 | 0.84 | 1.00 |
| SP | BCN | S | NF | 0.73 | 1.88 | 0.82 | 0.83 | 1.02 | 2.04 | 1.25 | 1.14 | 1.00 | 1.21 | 1.04 | 0.89 | 0.96 | 1.32 |
| SP | BCN | S | NF | 0.76 | 2.29 | 0.86 | 0.90 | 0.99 | 0.70 | 1.15 | 1.14 | 0.81 | 1.19 | 1.06 | 1.10 | 1.15 | 1.28 |
| SP | BCN | A | NF | 1.65 | 3.71 | 1.56 | 0.95 | 1.03 | 0.04 | 0.92 | 1.45 | 2.35 | 0.94 | 1.21 | 1.06 | 1.55 | 2.25 |
| SP | BCN | A | NF | 1.51 | 3.41 | 1.46 | 0.88 | 0.99 | 0.03 | 1.18 | 1.35 | 1.06 | 1.49 | 1.08 | 1.42 | 0.95 | 3.25 |
| SU | BCN | S | F | 1.94 | 7.60 | 2.56 | 3.99 | 1.16 | - | 1.43 | 3.56 | 2.93 | 1.31 | 0.97 | 1.54 | 2.30 | 1.64 |
| SU | BCN | S | F | 1.93 | 0.64 | 1.59 | 2.89 | 1.15 | - | 1.12 | 2.47 | 2.67 | 1.32 | 1.22 | 1.26 | 1.53 | 1.23 |
| SU | BCN | A | F | 1.36 | 4.17 | 1.53 | 3.18 | 0.92 | - | 1.01 | 3.32 | 4.49 | 1.49 | 1.65 | 1.26 | 2.93 | 2.36 |
| SU | BCN | A | F | 2.55 | 2.29 | 2.37 | 3.37 | 1.15 | - | 1.05 | 3.47 | 5.03 | 1.40 | 1.58 | 1.73 | 3.53 | 1.58 |
| SU | BCN | S | NF | 1.72 | 5.22 | 1.92 | 2.20 | 0.90 | 2.44 | 1.58 | 1.62 | 1.10 | 1.71 | 1.01 | 0.58 | 0.83 | 1.14 |
| SU | BCN | S | NF | 1.95 | 3.67 | 2.01 | 2.75 | 1.04 | 1.58 | 1.41 | 2.11 | 1.41 | 1.27 | 0.95 | 0.72 | 2.47 | 0.49 |
| SU | BCN | A | NF | 1.63 | 1.11 | 1.58 | 2.00 | 1.06 | 1.43 | 1.39 | 1.56 | 0.88 | 1.35 | 0.90 | 1.85 | 0.64 | 0.80 |
| SU | BCN | A | NF | 3.24 | 8.23 | 3.40 | 1.90 | 0.94 | 1.79 | 1.36 | 2.12 | 1.24 | 1.10 | 0.95 | 1.08 | 0.67 | 1.02 |
| SP | BLA | S | NF | 1.59 | 2.01 | 1.62 | 0.97 | 0.98 | 85.14 | 0.93 | 1.17 | 2.23 | 1.14 | 3.89 | 0.89 | 3.07 | 1.06 |
| SP | BLA | S | NF | 1.50 | 1.87 | 1.53 | 0.95 | 0.99 | 19.49 | 1.00 | 1.16 | 2.38 | 1.55 | 6.01 | 1.15 | 3.21 | 1.56 |
| SP | BLA | A | NF | 2.75 | 21.73 | 3.75 | 1.05 | 1.07 | 0.85 | 0.99 | 1.84 | 4.30 | 7.12 | 14.57 | 1.17 | 4.61 | 2.14 |
| SP | BLA | A | NF | 2.69 | 21.52 | 3.59 | 1.09 | 1.07 | 1.67 | 1.28 | 2.09 | 0.93 | 3.09 | 2.80 | 2.15 | 2.38 | 1.66 |
| SU | BLA | S | NF | 1.22 | 3.81 | 1.68 | 1.00 | 0.93 | 1.14 | 1.00 | 1.35 | 1.06 | 1.08 | 1.10 | 1.12 | 1.18 | 1.40 |
| SU | BLA | S | NF | 1.92 | 0.20 | 1.46 | 0.82 | 0.96 | 0.93 | 1.01 | 1.45 | 0.79 | 1.04 | 1.07 | 1.53 | 1.35 | 1.24 |
| SU | BLA | A | NF | 3.86 | 6.45 | 4.48 | 0.89 | 1.10 | 1.51 | 1.01 | 2.01 | 1.33 | 1.19 | 1.57 | 1.79 | 1.60 | 1.44 |
| SU | BLA | A | NF | 4.33 | 7.57 | 5.07 | 0.81 | 0.95 | 2.85 | 1.22 | 1.96 | 0.91 | 1.32 | 1.19 | 1.26 | 1.02 | 1.20 |
| SU | OFF | S | NF | 3.93 | 2.02 | 2.94 | 1.06 | 1.00 | 1.07 | 1.11 | 1.19 | 1.13 | 1.21 | 1.15 | 1.87 | 1.78 | 1.34 |
| SU | OFF | S | NF | 3.83 | 1.66 | 2.76 | 1.16 | 0.96 | 1.24 | 0.99 | 0.95 | 1.05 | 1.09 | 1.20 | 2.08 | 2.30 | 0.98 |
| SU | OFF | A | NF | 4.76 | 1.54 | 3.18 | 1.07 | 1.02 | 2.26 | 0.93 | 3.20 | 3.21 | 1.97 | 1.97 | 5.09 | 5.11 | 1.59 |
| SU | OFF | A | NF | 5.01 | 2.32 | 3.66 | 1.21 | 1.04 | 1.85 | 0.95 | 3.32 | 3.50 | 2.30 | 2.42 | 7.13 | 7.53 | 1.99 |
